# Effects ﻿of esketamine on patient-reported outcomes in major depressive disorder with active suicidal ideation and intent: a pooled analysis of two randomized phase 3 trials (ASPIRE I and ASPIRE II)

**DOI:** 10.1007/s11136-023-03451-9

**Published:** 2023-07-13

**Authors:** Carol Jamieson, Carla M. Canuso, Dawn F. Ionescu, Rosanne Lane, Xin Qiu, Heather Rozjabek, Patricio Molero, Dong-Jing Fu

**Affiliations:** 1 Janssen Research & Development, LLC, Milpitas, CA USA; 2 Janssen Research & Development, LLC, Titusville, NJ USA; 3 Janssen Research & Development, LLC, San Diego, CA USA; 4 Janssen Research & Development, LLC, Raritan, NJ USA; 5 Department of Psychiatry, Clinica Universidad de Navarra, Pamplona, Spain

**Keywords:** Esketamine, Major depressive disorder, Patient-reported outcomes, Pooled analysis, Suicidal ideation

## Abstract

**Purpose:**

To assess the effect of esketamine nasal spray on patient-reported outcomes (PROs) in patients with major depressive disorder having active suicidal ideation with intent (MDSI).

**Methods:**

Patient-level data from two phase 3 studies (ASPIRE I; ASPIRE II) of esketamine + standard of care (SOC) in patients (aged 18–64 years) with MDSI, were pooled. PROs were evaluated from baseline through end of the double-blind treatment phase (day 25). Outcome assessments included: Beck Hopelessness Scale (BHS), Quality of Life (QoL) in Depression Scale (QLDS), European QoL Group-5-Dimension-5-Level (EQ-5D-5L), and 9-item Treatment Satisfaction Questionnaire for Medication (TSQM-9). Changes in BHS and QLDS scores (baseline to day 25) were analyzed using a mixed-effects model for repeated measures (MMRM).

**Results:**

Pooled data for esketamine + SOC (n = 226; mean age: 40.5 years, 59.3% females) and placebo + SOC (n = 225; mean age: 39.6 years, 62.2% females) were analyzed. Mean ± SD change from baseline to day 25, esketamine + SOC vs placebo + SOC (least-square mean difference [95% CI] based on MMRM): BHS total score, − 7.4 ± 6.7 vs − 6.8 ± 6.5 [− 1.0 (− 2.23, 0.21)]; QLDS score, − 14.4 ± 11.5 vs − 12.2 ± 10.8 [− 3.1 (− 5.21, − 1.02)]. Relative risk (95% CI) of reporting perceived problems (slight to extreme) in EQ-5D-5L dimensions (day 25) in esketamine + SOC vs placebo + SOC: mobility [0.78 (0.50, 1.20)], self-care [0.83 (0.55, 1.27)], usual activities [0.87 (0.72, 1.05)], pain/discomfort [0.85 (0.69, 1.04)], and anxiety/depression [0.90 (0.80, 1.00)]. Mean ± SD changes from baseline in esketamine + SOC vs placebo + SOC for health status index: 0.23 ± 0.21 vs 0.19 ± 0.22; and for EQ-Visual Analogue Scale: 24.0 ± 27.2 vs 19.3 ± 24.4. At day 25, mean ± SD in domains of TSQM-9 scores in esketamine + SOC vs placebo + SOC were: effectiveness, 67.2 ± 25.3 vs 56.2 ± 26.8; global satisfaction, 69.9 ± 25.2 vs 56.3 ± 27.8; and convenience, 74.0 ± 19.4 vs 75.4 ± 18.7.

**Conclusion:**

These PRO data support the patient perspective of the effect associated with esketamine + SOC in improving health-related QoL in patients with MDSI.

**Trial registration**: ClinicalTrials.gov Identifier: ASPIRE I, NCT03039192 (Registration date: February 1, 2017); ASPIRE II, NCT03097133 (Registration date: March 31, 2017).

**Supplementary Information:**

The online version contains supplementary material available at 10.1007/s11136-023-03451-9.

## Introduction

Depression is a leading cause of disability affecting nearly 300 million people globally and is a major contributor to suicide deaths worldwide [1]. In the United States, major depressive disorder (MDD) affects 7.8% of the adult population annually [2], with an estimated 31% of MDD patients experiencing past-year suicidal ideation [3]. Prior studies have demonstrated that individuals with MDD and suicidal ideation are often associated with poorer health-related quality of life (HRQoL), greater work productivity loss and activity impairment than those without suicidal ideation [4, 5].

Current standard of care (SOC) includes inpatient psychiatric hospitalization and optimized oral antidepressant therapy for patients at risk for suicide [6, 7]. Initial hospitalization aims to provide a safe environment for evaluation and initiation of treatment during emergencies; however, the risks for suicide remain high following discharge [8, 9]. Furthermore, standard antidepressants usually require approximately one month or more for antidepressant effects to manifest, hence their utility in such situations remains limited [7, 10].

Esketamine nasal spray plus comprehensive SOC has been approved for treatment of depressive symptoms in patients with MDD having active suicidal ideation with intent by the US Food and Drug Administration, the European Medicines Agency, and other global health authorities. These approvals were based on efficacy and safety results from two identically designed, double-blind, randomized, placebo-controlled, multicenter phase 3 studies, ASPIRE I (NCT03039192) and ASPIRE II (NCT03097133) [11, 12]. In both studies, esketamine nasal spray plus SOC was associated with a rapid reduction in depressive symptoms of MDD in patients with active suicidal ideation with intent at 24 h after the first dose compared with a matched placebo nasal spray plus SOC, as assessed by clinician-rated outcome measures [11, 12]. The safety profile of esketamine in the high-risk patient population reported in these studies was consistent with the established safety profile of esketamine nasal spray [11, 12].

Clinically significant impairment in HRQoL, including perceived physical and mental functioning, are well documented among patients with MDD [13–18]. Patients with MDD reported greater limitations in physical, social, and role functioning, including work, household, and school activities than patients with many other chronic general medical conditions [19–21]. Patient-reported outcomes (PROs) were implemented in the ASPIRE clinical trials to capture some aspects of patient burden.

Inclusion of PROs in clinical trials also provides insights regarding unique patient perspectives, which can be used to help clinicians arrive at more informed treatment decisions, particularly for chronic, disabling conditions [22–24]. PROs play a crucial role in capturing the patient perspective of treatment and assist with examining the effects of treatment interventions in MDD and in predicting relapse [25, 26]. To obtain further insight into the efficacy of esketamine nasal spray, we evaluated its impact over time from a patient’s perspective through PROs using pooled data from the ASPIRE I and ASPIRE II trials.

## Methods

### Study design and participants

Data for this post hoc analysis were pooled from ASPIRE I and ASPIRE II, two identically designed, randomized, double-blind, placebo-controlled, multicenter, phase 3 studies that evaluated efficacy and safety of esketamine nasal spray vs matched placebo nasal spray co-administered with a newly optimized oral antidepressant therapy and initial hospitalization as SOC. The study design and methodology have been published [11, 12, 27]. In brief, an initial screening phase conducted within 48 h prior to day 1 dose was followed by a 4-week double-blind treatment phase wherein patients were randomized (1:1) to receive esketamine (84 mg) nasal spray plus SOC or placebo plus SOC twice weekly. The studies included patients (aged 18–64 years) with a diagnosis of MDD (as per Diagnostic and Statistical Manual of Mental Disorders, 5th edition [DSM-5] criteria) [28] without psychosis, as confirmed by the Mini International Neuropsychiatric Interview; current suicidal ideation with intent within 24 h prior to randomization, as confirmed by responding ‘Yes’ to the questions “Think about suicide?” and “Intend to act on thoughts of killing yourself?”; in need of acute psychiatric hospitalization due to imminent risk of suicide; and with a Montgomery–Åsberg Depression Rating Scale (MADRS) total score > 28 pre-dose on day 1.

Both trials were conducted in accordance with the ethical principles of the Declaration of Helsinki International Conference on Harmonization, Good Clinical Practice guidelines, and applicable regulatory requirements. All patients provided written informed consent. Study protocols and amendments were approved by independent review board or ethics committee for each study site.

### Patient-reported outcomes

The trials included the following PRO instruments that capture relevant concepts from the patients’ perspectives: The Beck Hopelessness Scale (BHS), the QoL in Depression Scale (QLDS), the European QoL Group, 5-Dimension, 5-Level (EQ-5D-5L), and the 9-item Treatment Satisfaction Questionnaire for Medication (TSQM-9).

All PRO measures were self-administered by the patients and data were collected using an electronic tablet. All PROs were evaluated from baseline through the end of the double-blind treatment phase (day 25).

#### Beck﻿ Hopelessness Scale [29, 30]

The BHS is a self-reported measure to assess one’s level of negative expectations or pessimism regarding the future. It consists of 20 true/false items that examine the respondent’s attitude over the past week by either endorsing a pessimistic statement or denying an optimistic statement. These items fall within 3 domains: 1. feelings about the future; 2. loss of motivation; and 3. future expectations. For every statement, each response is assigned a score of 0 or 1. The total BHS score is a sum of item responses and ranges from 0 to 20 (Minimal: 0–3, Mild: 4–8, Moderate: 9–14, and Severe: 15–20), with a higher score representing a higher level of hopelessness. BHS score of ≥ 9 have been found to be predictive of eventual suicide in depressed individuals with suicidal ideation [31–35].

#### Quality of Life in Depression Scale [36]

The QLDS is a disease specific, 34-item questionnaire designed to assess HRQoL in patients with MDD. Patients choose “true”/“not true” for each item based on their health “at the present time,” for a possible total score from 0 (good quality of life [QoL]) to 34 (poor QoL). A meaningful change threshold of 8 points has been suggested for depressed patients [37].

#### European Quality of Life Group, 5-Dimension, 5-Level [38]

The EQ-5D-5L, a standardized 2-part instrument for assessing HRQoL, consists of the EQ-5D-5L descriptive system and the EuroQol Visual Analogue Scale (EQ-VAS). EQ-5D-5L descriptive system has 5 dimensions of health (mobility, self-care, usual activities, pain/discomfort, and anxiety/depression) scored on five levels based on perceived problems (Level 1: none to Level 5: extreme). Patients selected an answer for each dimension considering the response that best matched his or her health “today”. Each dimension’s response was used to generate a health status index (HSI; 0 [dead] to 1 [full health]). Changes in HSI on the order of 0.03–0.07 are recognized as a threshold for meaningful change for an individual patient [39, 40]. Patients also self-rated their overall health status from 0 (worst health) to 100 (best health) on the EQ-VAS. Changes in EQ-VAS on the order of 7 to 10 are recognized as a threshold for meaningful change for an individual patient [41].

#### Treatment Satisfaction Questionnaire for Medication, 9 Items [42]

TSQM-9 is 9-item PRO instrument assessing patients’ satisfaction with the medication and covers the domains of effectiveness, convenience, and global satisfaction. Each domain is scored from 0 to 100 with lower scores indicating lower satisfaction. The recall period is “the last 2–3 weeks.”

### Statistical analyses

Descriptive statistics were provided for PROs collected at baseline and day 25 (TSQM-9 was collected at day 25 only).

The change from baseline to day 25 in BHS and QLDS scores was estimated using least squares (LS) means based on a mixed-effects model for repeated measures (MMRM) on observed case data with baseline score as covariate, and day, treatment, analysis center, SOC antidepressant treatment as randomized (antidepressant monotherapy, or antidepressant plus augmentation therapy) and day-by-treatment interaction as fixed effects and a random subject effect. The corresponding 95% confidence interval (CI) for the treatment difference were provided. Missing data were assumed to be missing at random.

Relative risks (95% CI) were provided to compare the risks of reporting perceived problems in each dimension of EQ-5D-5L (levels 2–5, indicating slight to extreme problems) at day 25 between two treatment groups. Proportion of patients with clinically meaningful improvement in PROs at day 25 were summarized, defined as change from baseline by the meaningful change threshold or greater for the QLDS, HSI, EQ-VAS at day 25, and proportion of patients with a BHS score of  < 9 (minimal or mild hopelessness) at day 25. Estimates of the treatment difference in proportions and 95% CIs were determined.

Since the PROs were not primary endpoints, sample size for each study was calculated using the primary endpoint (MADRS total score). Assuming an effect size of 0.45 for the change in MADRS total score between esketamine plus SOC and placebo plus SOC, a two-sided significance level of 0.05, and a drop-out rate at 24 h of 5%, approximately 112 patients were required to be randomly assigned to each treatment group to achieve 90% power.

The full efficacy analysis set for all PRO analyses included all randomized patients who received at least 1 dose of double-blind study medication and had both a baseline and a post-baseline evaluation for the MADRS total score or Clinical Global Impression–Severity of Suicidality–revised.

The SAS version 9.4 was used to perform the statistical data analysis in this study.

## Results

### Patient disposition and characteristics

The combined data from the ASPIRE I and ASPIRE II studies resulted in 451 patients with MDD with suicidal ideation included in the full efficacy analysis set, with 226 randomized to esketamine + SOC and 225 to placebo + SOC. In this pooled analysis, baseline demographics and clinical characteristics across treatment arms were similar (Table 1). Mean patient age in the esketamine + SOC and placebo + SOC groups was 40.5 and 39.6 years, and the proportion of women was 59.3% and 62.2%, respectively. Baseline scores for the PROs were also comparable (Table 1). Further details regarding the demographics, baseline clinical and psychiatric information have been published previously for this pooled population [27].

**Table 1:** Baseline demographic and clinical characteristics of patients in the ASPIRE I and ASPIRE II (pooled data)

Parameter	Esketamine + SOC (n = 226)	Placebo + SOC (n = 225)
Age, mean	40.5	39.6
Female, n (%)	134 (59.3)	140 (62.2)
Race, n (%)		
African American	11 (4.9)	15 (6.7)
Asian	29 (12.8)	30 (13.3)
Caucasian	169 (74.8)	161 (71.6)
Other/multiple	11 (4.9)	11 (4.9)
Not reported	6 (2.7)	8 (3.6)
Ethnicity, n (%)		
Hispanic or Latino	38 (16.8)	36 (16.0)
Not Hispanic or Latino	181 (80.1)	180 (80.0)
Unknown	1 (0.44)	1 (0.44)
BHS total score, mean (SD)	15.4 (4.2)	15.8 (4.3)
QLDS score, mean (SD)	27.0 (6.3)	27.0 (5.8)
Health status index, mean (SD)	0.56 (0.20)	0.56 (0.20)
EQ-VAS score, mean (SD)	40.1 (23.6)	40.2 (23.8)

*BHS* Beck Hopelessness Scale; *EQ-VAS* EuroQol Visual Analogue Scale; *QLDS* Quality of Life in Depression Scale; *SD* standard deviation; *SOC* standard of care

### Patient-reported outcomes

#### Beck Hopelessness Scale

**Fig. 1:**
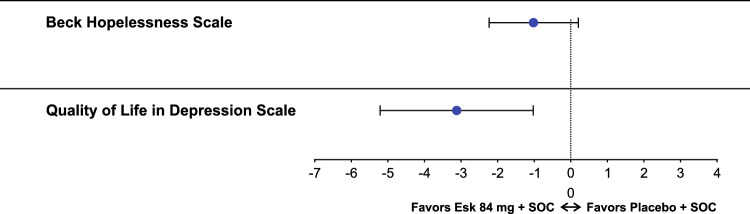
Mean difference in change from baseline at day 25 in BHS total score and QLDS total score (pooled data). The estimates and CIs are based on MMRM analysis. *BHS* Beck Hopelessness Scale; *CI* confidence interval; *ESK* esketamine; *LS* least squares; *MMRM* mixed-effects model for repeated measures; *QLDS* Quality of Life in Depression Scale; *SOC* standard of care

At baseline, mean BHS total scores were 15.4 for esketamine + SOC group and 15.8 for placebo + SOC group, indicating severe hopelessness. At day 25, the mean (SD) of the BHS total scores were 8.2 (6.6), and 8.9 (6.6) for patients treated with esketamine + SOC and placebo + SOC, respectively. The mean (SD) change from baseline to day 25 in BHS total score for esketamine + SOC and placebo + SOC groups was − 7.4 (6.7) vs − 6.8 (6.5) and difference of least squares mean (95% CI) was − 1.0 (− 2.23, 0.21) (Table 2; Fig. 1). Overall, 112 (49.6%) esketamine + SOC treated patients compared to 100 (44.4%) placebo + SOC treated patients had clinically significant improvement in BHS total score at day 25 (BHS < 9 minimal or mild hopelessness; difference in % [95% CI]: 5.1 [− 4.09, 14.31]; Table 3).

**Table 2:** Patient-reported outcomes in the ASPIRE I and ASPIRE II (pooled data)

	Esketamine + SOC (n = 226)		Placebo + SOC (n = 225)	
	n	Mean (SD)	n	Mean (SD)
BHS total score				
Baseline	224	15.4 (4.2)	224	15.8 (4.3)
Day 25	197	8.2 (6.6)	195	8.9 (6.6)
Change from baseline	196	− 7.4 (6.7)	194	− 6.8 (6.5)
Difference of LS means (95% CI)	− 1.0 (− 2.23; 0.21)			
QLDS score				
Baseline	225	27.0 (6.3)	224	27.0 (5.8)
Day 25	196	12.6 (11.3)	193	14.7 (10.8)
Change from baseline	196	− 14.4 (11.5)	192	− 12.2 (10.9)
Difference of LS means (95% CI)	− 3.1 (− 5.21; − 1.02)			
HSI				
Baseline	225	0.56 (0.20)	224	0.56 (0.20)
Day 25	196	0.79 (0.17)	193	0.76 (0.18)
Change from baseline	196	0.23 (0.21)	192	0.19 (0.22)
EQ-VAS score				
Baseline	225	40.1 (23.62)	224	40.2 (23.82)
Day 25	196	64.3 (22.34)	193	60.5 (22.41)
Change from baseline	196	24.0 (27.18)	192	19.3 (24.39)

For BHS and QLDS the LS means were based on MMRM model

*BHS* Beck Hopelessness Scale, *EQ-VAS* EuroQol Visual Analogue Scale; *HSI* Health Status Index; *LS* least squares; *MMRM *mixed-effects model for repeated measures; *QLDS* Quality of Life in Depression Scale; *SD* standard deviation;* SOC* standard of care

**Table 3:** Proportion of responders or proportion of patients with clinically meaningful improvement in patient-reported outcomes in the ASPIRE I and ASPIRE II (pooled data)

	Esketamine + SOC (n = 226)	Placebo + SOC (n = 225)
QLDS change from baseline to day 25 ≤ -8, n (%)	132 (58.4)	113 (50.2)
Difference in % (95% CI)	8.2 (− 0.98; 17.35)	
HSI change from baseline to day 25 ≥ 0.03, n (%)	168 (74.3)	147 (65.3)
Difference in % (95% CI)	9.0 (0.57; 17.43)	
HSI change from baseline to day 25 ≥ 0.07, n (%)	152 (67.3)	134 (59.6)
Difference in % (95% CI)	7.7 (− 1.16; 16.56)	
EQ-VAS change from baseline to day 25 ≥ 7, n (%)	139 (61.5)	126 (56.0)
Difference in % (95% CI)	5.5 (− 3.57; 14.58)	
EQ-VAS change from baseline to day 25 ≥ 10, n (%)	137 (60.6)	121 (53.8)
Difference in % (95% CI)	6.8 (− 2.27; 15.95)	
BHS at day 25 < 9, n (%)	112 (49.6)	100 (44.4)
Difference in % (95% CI)	5.1 (− 4.09; 14.31)	

The confidence intervals are based on Wald statistic

Patients who do not meet each criterion or discontinued treatment prior to the time point for any reason will not be considered to be in response or have made clinically meaningful improvements in PRO

QLDS score ranges from 0 to 34; a higher score indicates a more severe condition. A negative change indicates improvement

HSI ranges from − 0.148 to 0.949 and is anchored at 0 (health state valued equal to dead) and 1 (full health); a lower score indicates worse health. A positive change in score indicates improvement

VAS score ranges from 0 to 100; a lower score indicates worse health. A positive change in score indicates improvement

BHS total score ranges from 0 to 20; a higher score indicates a more severe condition

The confidence intervals are based on Wald statistic

*BHS* Beck Hopelessness Scale; *EQ-VAS* EuroQol Visual Analogue Scale; *HSI* Health Status Index; *LS* least squares; *PRO* patient-reported outcome; * QLDS* Quality of Life in Depression Scale; *SD* standard deviation; *SOC* standard of care;* VAS* Visual Analogue Scale

#### Quality of Life in Depression Scale

At baseline, the mean QLDS score was 27.0 for both treatment arms. At day 25, the mean (SD) of the QLDS scores were 12.6 (11.3) and 14.7 (10.8) for patients treated with esketamine + SOC and placebo + SOC, respectively. The mean (SD) change from baseline to day 25 in QLDS score for esketamine + SOC and placebo + SOC groups was − 14.4 (11.5) vs − 12.2 (10.8) and difference of least squares mean (95% CI) was − 3.1 (− 5.21, − 1.02) (Table 2; Fig. 1). At day 25, 132 (58.4%) patients from the esketamine + SOC group vs 113 (50.25%) patients from the placebo + SOC group had at least 8 points reduction in QLDS score from baseline (difference in % [95% CI]: 8.2 [− 0.98, 17.35]; Table 3).

#### European Quality of Life Group, 5-Dimension, 5-Level

**Fig. 2:**
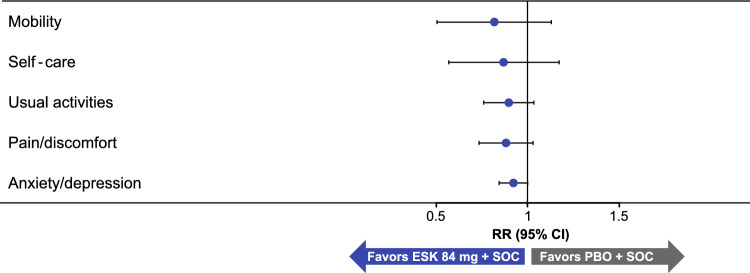
Relative risk of reporting slight problems or worse on each dimension of the EQ-5D-5L at day 25 (pooled data). *CI* confidence interval; *EQ-5D-5L* European Quality of Life Group, 5-Dimension, 5-Level; *ESK* esketamine; *PBO* placebo; *RR* relative risk; *SOC* standard of care

The relative risk (95% CI) of reporting perceived problems in EQ-5D-5L (levels 2–5, indicating slight to extreme problems) at day 25 in esketamine + SOC compared with placebo + SOC group in each dimension were: mobility, 0.78 (0.50, 1.20); self-care, 0.83 (0.55, 1.27); usual activities, 0.87 (0.72, 1.05); pain/discomfort, 0.85 (0.69, 1.04); anxiety/depression, 0.90 (0.80, 1.00) (Fig. 2). At day 25, for all dimensions (mobility, self-care, usual activities, pain/discomfort, and anxiety/depression) of EQ-5D-5L, a lesser proportion of patients treated with esketamine + SOC vs placebo + SOC reported having any perceived problems (slight [Level 2] to extreme problems [Level 5]) (Supplementary Fig. 1).

At baseline, the mean (SD) HSI was 0.56 (0.20) for both treatment arms. At day 25, the mean (SD) of the HSI were 0.79 (0.17) and 0.76 (0.18) for patients treated with esketamine + SOC and placebo + SOC, respectively. The mean (SD) change from baseline to day 25 in HSI for esketamine + SOC and placebo + SOC groups was 0.23 (0.21) vs 0.19 (0.22) (Table 2). At day 25, 168 (74.3%) patients treated with esketamine + SOC and 147 (65.3%) patients treated with placebo + SOC reported HSI change from baseline of ≥ 0.03. While in 152 (67.3%) and 134 (59.6%) patients treated with esketamine + SOC and placebo + SOC, respectively, a change of ≥ 0.07 was reported (Table 3).

At baseline, the mean EQ-VAS scores were 40.1 (23.6) for esketamine + SOC group and 40.2 (23.8) for placebo + SOC group. At day 25, the mean (SD) of the EQ-VAS scores were 64.3 (22.3) and 60.5 (22.4) for patients treated with esketamine + SOC and placebo + SOC, respectively. The mean (SD) change from baseline to day 25 in EQ-VAS score for esketamine + SOC and placebo + SOC groups was 24.0 (27.2) vs 19.3 (24.4) (Table 2), with 139 (61.5%) patients receiving esketamine + SOC and 126 (56.0%) patients receiving placebo + SOC reporting EQ-VAS change from baseline of ≥ 7 (Table 3). While in 137 (60.6%) and 121 (53.8%) patients treated with esketamine + SOC and placebo + SOC, respectively, a change of ≥ 10 was reported (Table 3).

#### Treatment Satisfaction Questionnaire for Medication, 9 Items

**Fig. 3:**
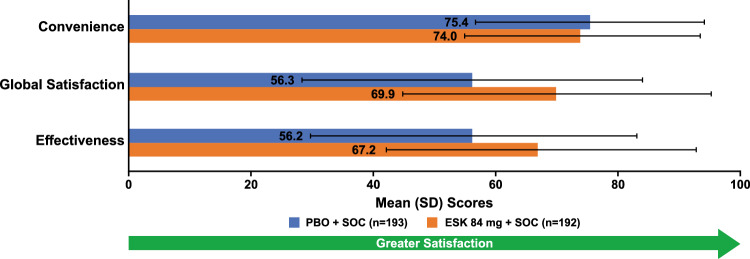
TSQM-9 scores at day 25 (pooled data). *ESK* esketamine; *PBO* placebo; *SD* standard deviation; *SOC* standard of care; *TSQM*-9 Treatment Satisfaction Questionnaire for Medication, 9 Items

The mean (SD) at day 25 in various domains of TSQM-9 scores in patients treated with esketamine + SOC vs placebo + SOC were: effectiveness, 67.2 (25.3) vs 56.2 (26.8); global satisfaction, 69.9 (25.2) vs 56.3 (27.8); convenience, 74.0 (19.4) vs 75.4 (18.7) (Fig. 3).

## Discussion

Both phase 3 global studies (ASPIRE I and ASPIRE II) have demonstrated clinically meaningful and statistically significant improvement in depressive symptoms evaluated as reduction in MADRS total score at 24 h post-first dose in MDD population with active suicidal ideation with intent [11, 12]. The results obtained from the recent pooled analysis were also consistent with those of the individual trials [27]. The current post hoc analysis of PROs supplements these efficacy results obtained using clinician-reported measures of depression. Overall, the findings of this analysis suggested that from the patient perspective (as assessed by PROs), esketamine + SOC treatment compared with placebo + SOC treatment led to numerically greater improvements in feelings of hopelessness assessed with the BHS, global satisfaction and effectiveness as assessed by the TSQM-9, and HRQoL as assessed with the EQ-5D-5L. Improvements in depression specific QoL as assessed with the QLDS showed more notable changes at day 25.

The patient-reported data collected via an online patient community platform, PatientsLikeMe, identified feelings of hopelessness, loneliness, anhedonia, and social anxiety to be significantly associated with suicidal ideation in patients with MDD [43]. Improvements in hopelessness, as measured by the BHS scores, was observed in both treatment groups. However, esketamine + SOC treatment showed numerically greater reduction in feelings of hopelessness prevalent among patients with active suicidal ideation and intent and numerically higher percentage of patients with scores of below 9 on the BHS. A greater proportion of patients showed improvements in QLDS (QLDS change from baseline ≤  − 8; Table 3) and EQ-5D-5L scores post-treatment with esketamine, suggesting an overall improvement in HRQoL. Patients’ satisfaction with the medication, as measured by TSQM-9 at day 25, provides an assessment of treatment effectiveness from the patient’s perspective, with numerically higher levels of satisfaction with effectiveness and global satisfaction among esketamine-treated patients than placebo-treated patients. There was no difference in their assessment of convenience, which is not surprising given that both treatment groups were taking study medication twice a week via intranasal administration.

Per recent FDA guidance [44], the collection of patient experience data (including information collected via PROs) is becoming increasingly important for the enhancement of regulatory decision making, in order to address patient needs. Here, we show that PROs supplement the use of traditional clinician rating scales to further support impact of esketamine in the treatment of patients with MDD with suicidal ideation and intent.

Some limitations of this study should be considered. Patients enrolled in clinical studies may differ from those in practice, so the generalizability of the study results may be limited particularly since patients in the ASPIRE I and ASPIRE II were provided comprehensive and optimized clinical care such as hospitalization and frequent clinical assessment which may have influenced PROs. The relationship with other sociodemographic variables, such as age, gender, race, or ethnicity, was not investigated. The post hoc nature of this analysis is another potential limitation.

In conclusion, these PRO data provide support for the patient perspective of effect associated with esketamine + SOC treatment in improving HRQoL in MDD patients with suicidal ideation and intent.

### Supplementary Information

Below is the link to the electronic supplementary material.

Supplementary file1 (PDF 443 KB)

## Data Availability

All data generated or analyzed during this study are included in this published article and its supplementary information files. The data sharing policy of Janssen Pharmaceutical Companies of Johnson & Johnson is available at https://www.janssen.com/clinical-trials/transparency. As noted on this site, requests for access to the study data can be submitted through Yale Open Data Access [YODA] Project site at http://yoda.yale.edu.

## Declarations

### Research involving human and animal participants

All procedures performed in studies involving human participants were in accordance with the International Council for Harmonization Good Clinical Practice Consolidated Guideline, the applicable local laws and regulatory requirements of the countries in which the trial was conducted, and with the principles of the Declaration of Helsinki. The findings of this study were reported in accordance with the CONSORT PRO guidelines for reporting patient-reported outcomes in randomized-controlled trials
