# Supplementary material for: Effects ﻿of esketamine on patient-reported outcomes in major depressive disorder with active suicidal ideation and intent: a pooled analysis of two randomized phase 3 trials (ASPIRE I and ASPIRE II)
Source: Qual Life Res. 2023 Jul 13;32(11):3053–61. doi: 10.1007/s11136-023-03451-9 (PMC10522733; doi:10.1007/s11136-023-03451-9)
Supplement: Supplementary file 1 — Supplementary file1 (PDF 443 KB) [file 11136_2023_3451_MOESM1_ESM.pdf]

Supplementary Fig. 1 Frequency distribution of patients reporting problems on each dimension of the EQ-5D-5L at baseline and day 25 (pooled data)

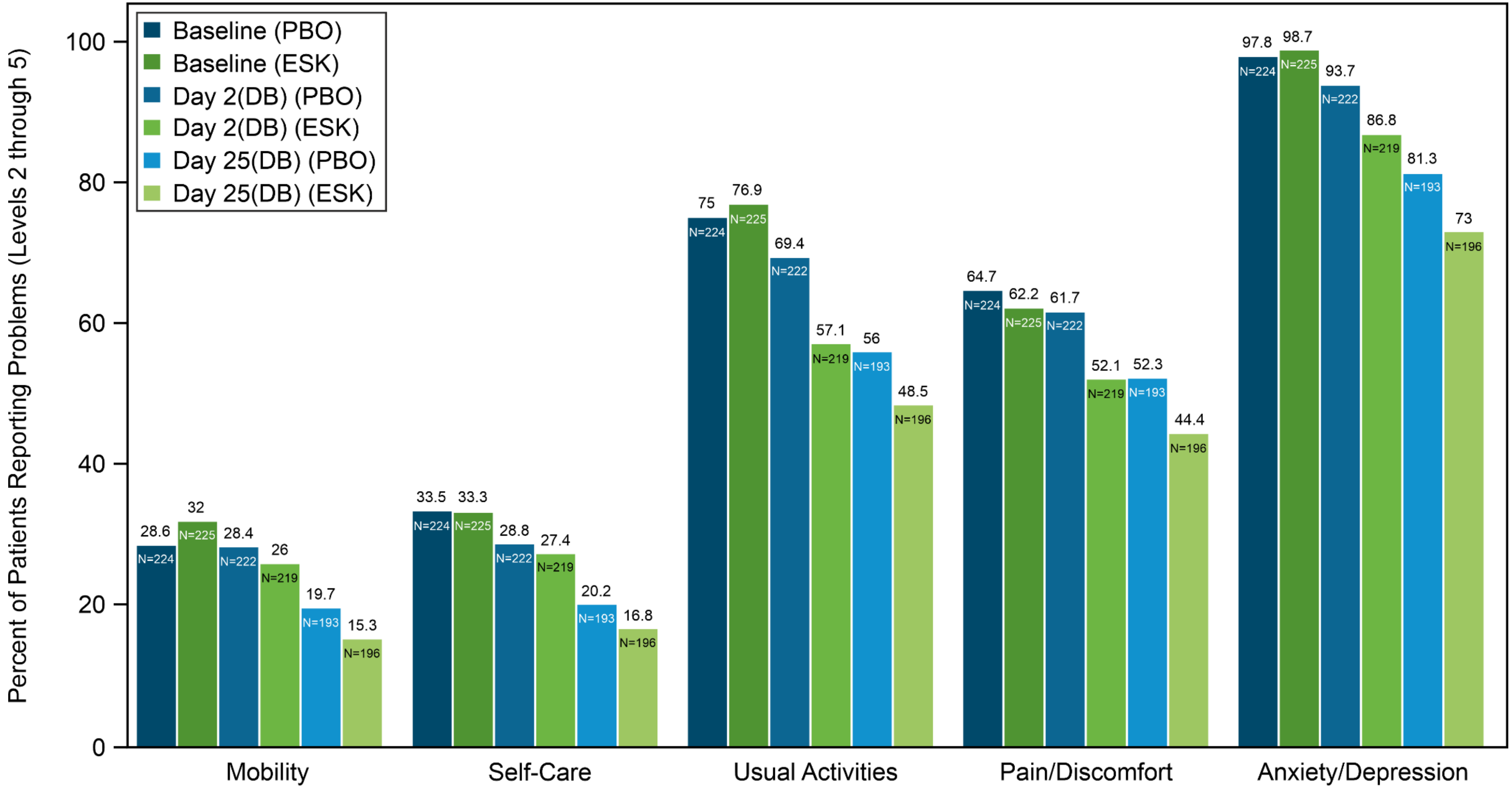

EQ-5D-5L, European Quality of Life Group, 5-Dimension, 5-Level; ESK, esketamine; PBO, placebo
